# First identification of *Angiostrongylus* spp. in *Lissachatina fulica* and *Cornu aspersum* in Antioquia, Colombia

**DOI:** 10.7705/biomedica.7051

**Published:** 2024-08-29

**Authors:** Ramón Gamarra-Rueda, Ricardo García, Diana C. Restrepo-Rodas, Janeth Pérez-García

**Affiliations:** 1 Facultad de Medicina Veterinaria y Zootecnia, Universidad CES, Medellín, Colombia Universidad CES Facultad de Medicina Veterinaria y Zootecnia Universidad CES Medellín Colombia; 2 Laboratorio de Diagnóstico Veterinario, Instituto Colombiano de Medicina Tropical - ICMT, Sabaneta, Colombia Instituto Colombiano de Medicina Tropical - ICMT Laboratorio de Diagnóstico Veterinario Instituto Colombiano de Medicina Tropical - ICMT Sabaneta Colombia; 3 Departamento de Conservación y Bienestar Animal, Parque de la Conservación, Medellín, Colombia Parque de la Conservación Departamento de Conservación y Bienestar Animal Parque de la Conservación Medellín Colombia

**Keywords:** Angiostrongylus, *Strongylida* infections, snails, Mollusca, zoonoses, Colombia, Angiostrongylus, infecciones por *Strongylida*, caracoles, moluscos, zoonosis, Colombia

## Abstract

**Introduction.:**

Abdominal and neural angiostrongyliasis caused by *Angiostrongylus costaricensis* and *A. cantonensis,* respectively, are zoonotic diseases involving snails as intermediate hosts. Colombia has already reported human cases, and the increasing distribution of *Lissachatina fulica* and *Cornu aspersum* raises public health concerns due to the potential risk of disease transmission in areas where parasites and hosts coexist.

**Objective.:**

To identify the presence of *Angiostrongylus* spp. in snail species *L. fulica* and *C. aspersum* in Antioquia, Colombia.

**Materials and methods.:**

This prospective cross-sectional study had a population of 5,855 *L. fulica* and *C. aspersum* snails captured in the ten towns of the Valle de Aburrá (Antioquia, Colombia), 169 samples were collected in 28 sampling points. Lung tissues of the collected snails were dissected and analyzed to detect *Angiostrongylus* spp*.* through molecular techniques.

**Results.:**

*Angiostrongylus* spp. were identified in both *L. fulica* and *C. aspersum. Angiostrongylus costaricensis* was detected in 18 pooled prevalence of 30% (95% CI = 19.2-43.3), and Medellín was the municipality with the highest number of positive samples (33.3%). Seventy-two-point-two percent of the positive places reported the presence of rodents. None of the tests were positive for *A. cantonensis*.

**Conclusion.:**

Our findings provide important insights into the epidemiology and distribution of *Angiostrongylus* spp*.* in Antioquia, Colombia. The identification of these parasitic nematodes in *L. fulica* and *C. aspersum* highlights the potential role of these snails as intermediate hosts in the transmission of *Angiostrongylus* spp. infections in the Valle de Aburrá, with implications for human and veterinary health.

## Resumen

**Introducción.:**

La angiostrongiliasis abdominal y neura -causadas por *Angiostrongylus costaricensis* y *A. cantonensis,* respectivamente- son zoonosis que involucran caracoles como huéspedes intermediarios. Colombia ya ha reportado casos en humanos y la ampliación de la distribución de *Lissachatina fulica* y *Cornu aspersum* aumenta la preocupación en salud pública debido al riesgo potencial de transmisión en áreas donde los parásitos y sus huéspedes coexisten.

**Objetivo.:**

Identificar la presencia de *Angiostrongylus* spp*.* en caracoles de las especies *L. fulica* y *C. aspersum* en Antioquia (Colombia).

**Materiales y métodos.:**

Se llevó a cabo un estudio transversal prospectivo con una población de 5.855 caracoles de *L. fulica* o *C. aspersum,* capturados en diez ciudades del valle de Aburrá; 169 muestras fueron recolectadas en 28 puntos de muestreo. Se disecaron los tejidos pulmonares de los caracoles y se emplearon técnicas moleculares para detectar la presencia de *Angiostrongylus* spp*.*

**Resultados.:**

*Angiostrongylus costaricensis* fue detectado en 18 muestras agrupadas (30 %; IC_95%_: 19,2-43,3), tanto en *L. fulica* como en *C. aspersum*. Medellín fue el municipio con el mayor número de muestras positivas (33,3 %). El 72,2 % de los lugares positivos reportaron la presencia de roedores. Ninguna de las pruebas fue positiva para *A. cantonensis.*

**Conclusión.:**

Estos hallazgos brindan información importante sobre la distribución de *Angiostrongylus* spp*.* en Antioquia (Colombia). La identificación de estos nemátodos en *L. fulica* y *C. aspersum* resalta el papel potencial de estos caracoles como huéspedes intermediarios en la transmisión de infecciones por *Angiostrongylus* en el valle de Aburrá, con implicaciones para la salud humana y veterinaria.

*Angiostrongylus* spp. are nematodes infecting humans and animals. These parasites are commonly found in rodents but can also reside in snails like *Cornu aspersum* (common garden snail) and *Lissachatina fulica* (giant African land snail) ^1^. *Angiostrongylus* spp*.* are endemic to Asia, the Pacific Islands (Hawaii, Vanuatu and Thailand), Australia, and the Caribbean islands. These parasites cause zoonotic diseases in humans: abdominal angiostrongyliasis caused by *A. costaricensis* and neural angiostrongyliasis caused by *A. cantonensis*. Recent human cases of the disease have been reported in various countries such as the United States, Spain, Ecuador, Brazil, Martinique (French West Indies), Venezuela and Colombia ^1^^-^^5^. The first case of human infection in Colombia was detected in 1979, with a total of eight events reported by 2020 ^6^.

The parasite life cycle begins with rats as definitive hosts ingesting it. Then, *Angiostrongylus* spp. penetrates the digestive system, enters the bloodstream, and migrates to the central nervous system until the parasite reaches the sub-adult stage. Finally, it migrates to the pulmonary arteries, depositing eggs that migrate to the lungs. In *A. costaricensis,* oviposition and hatching occur in the ileum. Hatched immature larvae migrate to the trachea, get ingested, and pass through the digestive system before being excreted. Snails become intermediate hosts by ingesting fecal material from infected rats containing first-stage larvae (L1) of *Angiostrongylus* spp. The parasite reaches its final infective stage, known as L_3_ in the snail. A new rat ingesting the infected snail allows the infective larvae to migrate via the bloodstream and initiate a new cycle. Humans become accidental hosts by the consumption of raw or undercooked snails or slugs ^7^. Additionally, *A. costaricensis* can infect dogs making them possible parasite reservoirs ^8^^,^^9^.

The giant African snail has been reported in more than 70% of Colombia’s departments in less than a decade, particularly in urban areas, where it finds refuge ^10^^-^^12^. In 2017, Cordoba *et al*. reported the presence of three parasitic nematode genera -*Angiostrongylus, Aelurostrongylus, and Strongyluris-* in giant African snails collected from nine towns of Valle del Cauca (Colombia): Jamundí, Cali, Palmira, Buga, Tuluá, Bugalagrande, Cartago, Dagua, and Buenaventura. However, they did not confirm the specific presence of *A. cantonensis*^6^^,^^13^^,^^14^.

Information on the prevalence of these parasites in snail intermediate hosts is scarce in Colombia. The disease is transmitted to humans through animal handling and fomites contaminated with snail secretions. Therefore, circulating *Angiostrongylus* spp.identification would determine the risk and allow the establishment of control measures for the disease and efficient protection of the intermediate snail host. While current Colombian regulations aim to control the African snail population ^15^, parasite recognition will allow the establishment of more radical control measures and reduce the risk of human infections. This study aimed to assess the presence of *Angiostrongylus* spp. in *C. aspersum* and *L. fulica* within the metropolitan area of Valle de Aburrá in 2022.

## Materials and methods

### Type of study and sample size

We conducted a prospective, cross-sectional, descriptive design. Data were collected from 28 sampling points distributed in ten towns of the metropolitan area from Valle de Aburrá: Barbosa, Bello, Caldas, Copacabana, Envigado, Girardota, Itagüí, La Estrella, Medellín, and Sabaneta. Due to resource limitations, we included a convenience sample size.

### Area of study

Valle de Aburrá exhibits significant altitudinal variation, with its major towns positioned between 1,400 meters and 1,600 meters above sea level, approximately. The region has a warm climate with relatively stable yearround temperatures ranging from 18 °C to 28 °C. Rainfall follows a bimodal pattern with a wet season from April to November and a drier season from December to March.

This region encompasses diverse urban and rural environments with unique climatic characteristics. Barbosa, situated in the northern region, is surrounded by moderate rainfall and rolling terrain. Girardota, a primarily rural area undergoing recent urbanization, receives high precipitation and has hilly topography. Itagüí, characterized by warm and humid conditions, exhibits variation in elevation. Bello, also situated to the south, shares similar conditions to Itagüí. Copacabana experiences a tropical climate with a well-defined wet and dry season. To the south, Envigado features a warm and temperate climate. La Estrella, also in the south, is characterized by flat terrain and warm, moderately rainy weather. Caldas, a predominantly residential area in the southwest, possesses flat terrain and a moderate climate. Sabaneta features a warm and temperate climate. Lastly, Medellín, the largest city in the region, resides in a mountain-enclosed valley, resulting in a subtropical mountainous climate with moderate temperatures.

### Snail sampling and specimen preparation

Sampling sites were chosen based on reports from the local community. Collection and identification of the species were carried out by the environmental authorities (*Área Metropolitana del Valle de Aburrá*). The collection period was from October 2022 to January 2023. Animals were transported to the *Parque de la Conservación*, where they were anesthetized with 5% ethanol, followed by euthanasia via immersion in 95% ethanol, according to the recommendations of the “Guide for euthanasia of animals” of the American Veterinary Medical Association 2020 ^16^.

The shells were separated from the soft tissues. For small individuals, the entire tissue was collected, and for larger snails, a portion of the mantle, foot, and lungs were excised. A total of 10 g was obtained per animal. Samples were placed in screw-cap urine cytochemical jars, kept refrigerated at approximately 4 °C, and transported daily to the laboratory at *Instituto Colombiano de Medicina Tropical* (Sabaneta, Colombia). There, samples were preserved at -20 °C until DNA extraction.

### DNA extraction

The DNA extraction was performed from 200 μg of the samples with the QIAGEN DNeasy Blood and Tissue Spin Column Kit®, following the manufacturer’s protocol. Each gram of macerated tissue was homogenized in 24 ml of lysis buffer (0.1 M Tris, 1 M EDTA, 0.01 M NaCl, 0.5% sodium dodecyl sulfate), combined with six to eight beads for 3 minutes; then, 150 μg of proteinase K were added to the mix and incubated at 56 °C for 1 to 48 hours, vortexing every hour until obtaining a homogeneous solution.

### Molecular testing

A multiple real-time PCR (qPCR) was performed to identify *Angiostrongylus* genus using the primer forward AcanITS1F (5’-TTC ATG GAT GGC GAA CTG ATA G-3’) and reverse AcanITS1R (5’-GCG CCC ATT GAA ACA TTA TAC TT-3’) to amplify a fragment detected by the probe AcanP (6-FAM-ATC GCA TAT CTA CTA TAC GCA TGT GAC ACC TG-BHQ1).

The PCR tests were carried out with the following thermal profile: reverse transcription step at 50 °C for 2 minutes, followed by 40 cycles of amplification with initial denaturation at 95 °C for 30 s, alignment and extension at 60 °C for 30 s, and final step at 56 °C for 2 minutes.

### Statistical analysis

The data were explored using the R program. Graphs and descriptive measures were generated for each variable: snail species, city, area type, presence of snails at the sampling site, and presence of rodents at the sampling site. The chi square test or Fisher’s test was calculated as a measure of association when necessary. A Pearson correlation test was applied to determine the relationship between positivity and the number of positive points by geographic location. The positivity frequency was calculated with a 95% confidence interval. Statistical significance was set at a p value less than 0.05.

### Ethical considerations

The project was approved by the *Comité Institucional de Cuidado y Uso de Animales de Experimentación* (CICUA) of the *Universidad CES* (approved by the minute number 27 of December 14, 2021, with project Code 65) and the ethics committee of the *Parque de la Conservación* (approved by the minute number 2 of November 30, 2021).

## Results

### Characteristics of snail sampling sites

**Figure 1:**
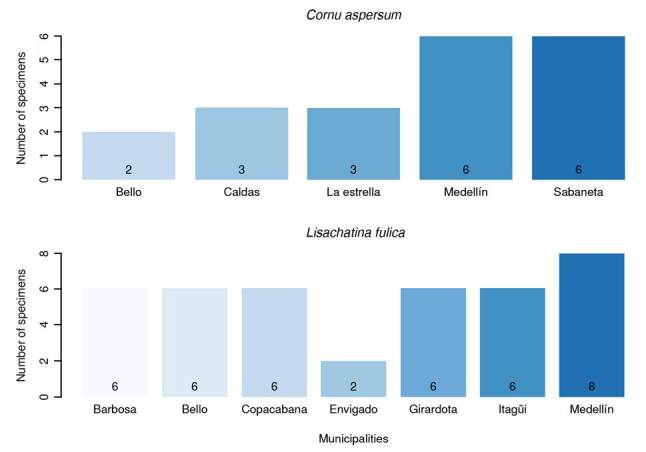
Number of samples by municipality in Valle de Aburrá (Antioquia)

Due to limited financial resources, only 169 individuals were sampled at 28 sampling points, distributed as follows: Medellín (28.57%; n = 8 points), Bello (14.28%; n = 4 points), Barbosa, Copacabana, Girardota, and Itagüí (10.71%; n = 3 points each) and Caldas, Envigado, Sabaneta, and La Estrella (3.57%; n = 1 point each). The distribution by collected species in each town is presented in figure 1.

Each sampling point yielded individuals of only one species. *L. fulica* (75%; n = 21 points) was the dominant species, followed by *C. aspersum* (25%; n = 7 points). Most sampling points (78.57%; n = 22 points) were in urban areas. There was no statistically significant association between area type (urban vs. rural) and the number of snails observed (p = 0.2300). Ten points (35.71%) presented a low quantity of snails, while the remaining eight (28.57%) had a higher abundance.

**Figure 2:**
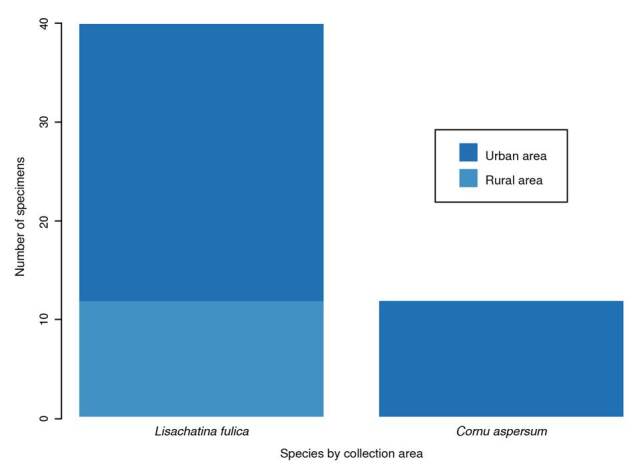
Distribution of the individuals included in the study by species and collection area in Valle de Aburrá (Antioquia)

Most samples were collected from urban areas, as shown in figure 2. No specimens of *L. fulica* were collected in rural areas. There was no statistically significant association between the collected species and the area type (p = 0.2875). Of the places where *L. fulica* was captured, 80.95% (n = 17) reported the presence of rodents, compared to only 42.85% (n = 3) of the sites where *C. aspersum* was collected. However, there was no association between the collected species and the presence of rodents (p = 0.1473).

### Presence of angiostrongyliasis

A total of 60 qPCR tests were performed by pooling two to three individuals per sample of the 169 animals. The proportion of pools positive for *A. costaricensis* was 30% (95% CI = 19.2-43.3), corresponding to 18 positive pools. None of the samples tested was positive for *A. cantonensis.* Of the sites with *C. aspersum*, 46.15% (n = 6 points) were positive, while 53.84% (n = 7 points) of the sites with *L. fulica* were positive for *A. costaricensis.* This difference between the snail species and the study site was statistically significant (p = 0.0286).

In addition, some environmental factors potentially associated with the presence of *A. costaricensis* revealed no statistically significant associations, as shown in table 1.

**Table 1:** Characteristics of study individuals according to the presence of *Angiostrongylus costaricensis* by qPCR, Valle de Aburrá (Antioquia, 2021)

Variable		Positive		Negative		p
		n	%	n	%	
Species						
	*Lissachatina fulica*	9	50.00	31	73.80	0.1352
	*Cornu aspersum*	9	50.00	11	26.19	
Towns						
	Barbosa	3	16.67	3	7.14	0.4003*
	Bello	3	16.67	5	11.90	
	Caldas	2	11.11	1	2.38	
	Copacabana	0	0.00	6	14.29	
	Envigado	0	0.00	2	4.76	
	Girardota	1	5.56	5	11.90	
	Itagüí	1	5.56	5	11.90	
	La Estrella	0	0.00	3	7.14	
	Medellín	6	33.33	8	19.05	
	Sabaneta	2	1111	4	9.52	
Zone						
	Urban	14	77.77	34	80.95	0.7400*
	Rural	4	22.22	8	19.04	
Presence of snails at sampling site						
	High	4	22.22	14	33.33	0.6250*
	Medium	6	33.33	10	23.81	
	Low	7	38.88	17	40.47	
	Accidental	1	5.55	1	2.38	
Presence of rodent at sampling site						
	Yes	13	72.22	27	64.28	0.7659*
	No	5	27.77	15 35.71		
Total		18 100		42	100	

*Fisher exact test

**Figure 3:**
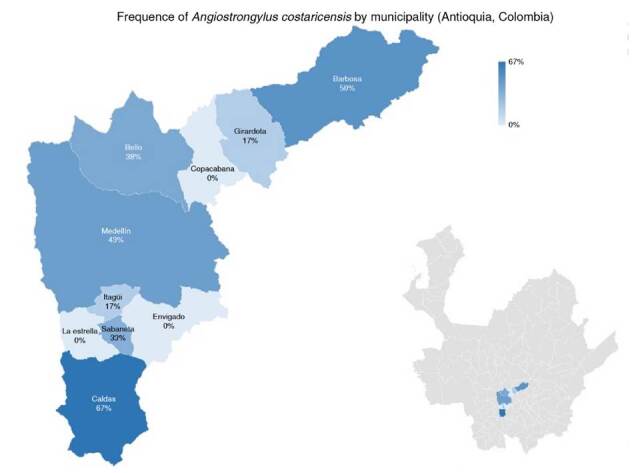
Frequence of Angiostrongylus costaricensis by municipality (Antioquia, Colombia)

Medellin had the highest number of positive samples (33.3%; n = 6), the highest number of positive samples from urban areas (77.77%; n = 14) and the places with a lower number of snails. The presence of rodents in the sampling sites and positive results in snails for *A. costaricensis* was 72% of all the sites included in the study (n = 13). Finally, no correlation was found between the number of snails collected and the number of sampling sites positive for *A. costaricensis* (p = 0.4408). The geographical distribution of snails infected with *A. costaricensis* in the metropolitan area of Valle de Aburrá is represented in figure 3.

## Discussion

This study found 30% positive pooled samples of *Angiostrongylus* spp. in snails across ten towns of the metropolitan area of Valle de Aburrá. This value is within the broad range of prevalence reported in mollusks across various geographical locations, such as Brazil, Ecuador, Egypt, and China, varying from 0.2 to 93.4% ^3^^,^^17^^-^^19^. These countries might share similarities influencing the transmission of *Angiostrongylus* spp*.,* such as climate variability, ecological diversity, and host availability.

In Colombia, few studies have described the presence of the parasite in African snails ^20^; Giraldo *et al.* in 2018 confirmed the presence of *A. cantonensis* in *L. fulica*’s lung tissue from Buenaventura (Valle del Cauca) by qPCR ^12^. This method is considered a direct test to identify *Angiostrongylus* presence and should be used for parasite detection during the transmission cycle. Through this study, it was possible to standardize the qPCR for continuous active epidemiological surveillance of the parasite.

The angiostrongyliasis prevalence or *Angiostrongylus* spp presence in the human population of Antioquia, in intermediate or final hosts, such as rodents, remains unknown. A study conducted in different departments of Colombia in 2019 reported a 3.9% frequency of *Angiostrongylus vasorum* in *L. fulica* but excluded the towns in this study and did not describe the presence of *A. costaricensis*^21^*.* The parasite presence should be considered an epidemiological alert for health authorities considering transmission routes, such as ingestion of contaminated vegetables, fruits, or legumes with *Angiostrongylus* spp.

Recognized cases of the disease are scarce in Colombia. However, *A. costaricensis* has been reported in departments like Caquetá, Huila, Putumayo, Tolima, Valle, and Vaupés, but no report is from the studied towns ^14^. This study aimed to determine the presence of *Angiostrongylus* spp. in *C. aspersum* and *L. fulica* in ten towns of the metropolitan area of Valle de Aburrá.

Despite the low sample sizes per municipality, the statistically significant difference in infection rates and collection municipality shows the need for deeper analysis of parasite abundance across the metropolitan area of the Valle de Aburrá due to its potential public health concern. Additionally, the higher number of *L. fulica* collected in urban areas reflects environmental awareness programs that have been carried out by local authorities but may not accurately represent the actual distribution of both species in the territory.

The reported association between the presence of rodents and positive snails suggests that the disease cycle may be in an enzootic phase. However, the imminent risk could be determined by incorporating rodents into the surveillance program about the exotic snails’ impact on health in the Valle de Aburrá, performing abundance studies of both snail species and determining angiostrongyliasis prevalence by systematic sampling per municipality. These studies should provide more accurate data and identify risk characteristics at specific sites.

Future studies should further investigate the eco-epidemiological role of snails, rats, parasites, and human communities in proximity. The epidemiology of angiostrongyliasis is complex and requires recognizing risk factors associated with the geographical conditions where *Angiostrongylus* spp. has been identified to implement effective measures for the prevention of human cases ^22^. This is crucial in Valle de Aburrá because of the high fauna biodiversity, microecosystems, water sources, land use patterns, and human settlements in each city.

This work is the first one reporting *A. costaricensis* in snails from seven of the ten studied towns in the Valle de Aburrá in Colombia. Our findings should be considered an epidemiological alert by the authorities to adapt protocols for managing and preventing infestation by invasive species like the African snail *L. fulica* and the subsequent transmission of disease-causing parasites to humans.
